# The Pathogenicity of *Fusobacterium nucleatum* Modulated by Dietary Fibers—A Possible Missing Link between the Dietary Composition and the Risk of Colorectal Cancer

**DOI:** 10.3390/microorganisms11082004

**Published:** 2023-08-03

**Authors:** Sadia Nawab, Qelger Bao, Lin-Hua Ji, Qian Luo, Xiang Fu, Shuxuan Fan, Zixin Deng, Wei Ma

**Affiliations:** 1 State Key Laboratory of Microbial Metabolism and School of Life Sciences and Biotechnology, Shanghai Jiao Tong University, 800 Dongchuan Road, Shanghai 200240, China; 2 Department of Gastrointestinal Surgery, Ren Ji Hospital, School of Medicine, Shanghai Jiao Tong University, 145 Middle Shandong Road, Shanghai 200001, China

**Keywords:** *Fusobacterium nucleatum*, butyrate, polydextrose, fibersol-2, colorectal cancer, dietary fibers

## Abstract

The dietary composition has been approved to be strongly associated with the risk of colorectal cancer (CRC), one of the most serious malignancies worldwide, through regulating the gut microbiota structure, thereby influencing the homeostasis of colonic epithelial cells by producing carcinogens, i.e., ammonia or antitumor metabolites, like butyrate. Though butyrate-producing *Fusobacterium nucleatum* has been considered a potential tumor driver associated with chemotherapy resistance and poor prognosis in CRC, it was more frequently identified in the gut microbiota of healthy individuals rather than CRC tumor tissues. First, within the concentration range tested, the fermentation broth of *F. nucleatum* exhibited no significant effects on Caco-2 and NCM460 cells viability except for a notable up-regulation of the expression of *TLR4* (30.70%, *p* < 0.0001) and *Myc* (47.67%, *p* = 0.021) and genes encoding proinflammatory cytokines including *IL1B* (197.57%, *p* < 0.0001), *IL6* (1704.51%, *p* < 0.0001), and *IL8* (897.05%, *p* < 0.0001) in Caco-2 cells exclusively. Although no marked effects of polydextrose or fibersol-2 on the growth of *F. nucleatum*, Caco-2 and NCM460 cells were observed, once culture media supplemented with polydextrose or fibersol-2, the corresponding fermentation broths of *F. nucleatum* significantly inhibited the growth of Caco-2 cells up to 48.90% (*p* = 0.0003, 72 h, 10%) and 52.96% (*p* = 0.0002, 72 h, 10%), respectively in a dose-dependent manner. These two kinds of fibers considerably promoted butyrate production of *F. nucleatum* up to 205.67% (*p* < 0.0001, 6% polydextrose at 24 h) and 153.46% (*p* = 0.0002, 6% fibersol-2 at 12 h), which explained why and how the fermentation broths of *F. nucleatum* cultured with fibers suppressing the growth of Caco-2 cells. Above findings indicated that dietary fiber determined *F. nucleatum* to be a carcinogenic or antitumor bacterium, and *F. nucleatum* played an important role in the association between the dietary composition, primarily the content of dietary fibers, and the risk of CRC.

## 1. Introduction

The incidence and mortality of CRC [1,2], one of the leading global health threats, have experienced a substantial increase in Asia [3] over the past decades, accompanying economic development and lifestyle changes [4]. For example, from the 1980s to the 2010s, Chinese people’s consumption of high protein and fatty foods, i.e., meat and fish was strikingly increased by 167% (from 60.7 to 162.4 g/day), while the intake of dietary fiber-rich food, such as cereals, tubers, beans, and vegetables, was declined by 34% (from 1056.5 to 694.1 g/day) [5]. Therefore, CRC incidence and mortality rate have become the second and fourth highest, respectively, among all kinds of tumors in China now, with a notable 126% increase in incidence and a mean annual increase of 9.5% [6] between 2011 to 2020. However, the mechanism behind the clear association between CRC and dietary structure has not been elucidated.

Numerous studies have provided evidence to support the significant effect of dietary composition on the structure of the gut microbiota [7,8]; for instance, one dietary intervention study demonstrated a significant increase in the abundance of *F. nucleatum* following a transition from a high-fiber, low-fat prudent-style diet to a low-fiber, and high-fat diet [9]. A specific community of gut microbiota could influence the homeostasis of colonic epithelial cells by producing carcinogenic [10] or antitumor metabolites [11].

Currently, the prevailing consensus is that short-chain fatty acids, represented by butyrate, were produced by commensal butyrate-producing bacteria in the large intestine [12,13,14] through fermentation of non-digestible dietary fibers [15] like polydextrose (PDX) [16] and fibersol-2 (FS2) [17]. Butyrate plays a critical role in maintaining gut epithelial cell homeostasis as their primary energy source, and it could inhibit CRC cells growth and promotes apoptosis by modulating the inhibition of histone deacetylases (HDAC) and NF-kB proinflammatory signaling pathways [18,19]. Interestingly, among four butyrate biosynthesis pathways, the pyruvate pathway [20] was predominantly identified in commensals bacteria, e.g., *Ruminococcaceae* [21], *Roseburia* [22], and *Lachnospiraceae* [23]; amino acids degrading pathways like 4-aminobutyrate [24] and lysine [25] were generally observed in *Porphyromonas gingivalis* [26], *Megasphaera* [27], *Clostridioides difficile* [28], and other carcinogenesis bacteria [29], while the glutarate pathway [30] was identified in both commensal and pathogenic bacteria [31], suggesting that the significant diversity of substrates, end-products, and side-products from different butyrate-producing pathways [32] might potentially impact the overall health of hosts and patients’ prognosis [33].

On one side, *F. nucleatum* has been extensively detected in CRCs [34,35], breast [36], lung [37] and other tumor tissues, with its higher abundance in cancerous tissue associated with chemotherapy failure and poor prognosis in CRC [38,39], and an experiment on *Apc*^−/+^ mice suggested its presence linked to CRC tumorigenesis [40] through activation of the β-catenin pathway via FadA adhesin binding to E-cadherin on host cells [41]. However, on the other side, *F. nucleatum* was identified in a mere 13% of CRC tumor tissues [34], yet it exhibited frequent occurrences in both the oral cavity [42] and the healthy individuals’ gut microbiota [43,44]. Furthermore, frequent observations of *F. nucleatum*, a bacterium with carcinogenic properties, actively synthesizing butyrate [45,46,47] has perplexed researchers for many years, posing a significant scientific challenge since butyrate has been conventionally recognized as an anti-inflammatory compound for an extended period [48]. However, *F. nucleatum* was experimentally proved to produce butyrate mainly utilizing amino acids [35] by lysine [45] and glutarate pathways [46,49], along with a 4-aminobutyrate pathway which was recently predicted through bioinformatics analysis [31,50].

Considering the ongoing controversies surrounding both the bioactivity of butyrate in a tumor, like CRC [51] and the precise role of *F. nucleatum* in CRC [52], the relationship between dietary fibers, *F. nucleatum*’s metabolites, and CRC was worth further study.

## 2. Materials and Methods

### 2.1. Strains and Culture

*F. nucleatum* subsp. *nucleatum* ATCC 25586 was from the preservation in our laboratory and cultured in BHI medium (Difco Laboratories, Franklin Lakes, NJ, USA) in the 85% N_2_, 10% H_2_, and 5% CO_2_ humidified anaerobic chamber (SHELLAB, Cornelius, OR, USA) at 37 °C. Overnight bacterial culture was used to anaerobically inoculate (1%) fresh BHI cultures with certain concentrations of dietary fibers and cultured for 48 h at 37 °C without agitation, and OD_600_ of culture was assayed using BioPhotometer (Eppendorf, Hamburg, Germany) at designated time points. Fermentation broths of *F. nucleatum* supplemented with sterile polydextrose (Danisco, Beaminster, UK) and fibersol-2 (Matsutani, Elk Grove Village, IL, USA) were collected when bacteria grew to OD_600_ = 1.0. The filter-sterilized supernatant was used for further cell experiments and GC-MS analysis.

### 2.2. Cell Lines and Cell Culture

Caco-2 (CRC) and NCM460 (normal) cells were from our laboratory’s stock. Caco-2 cells were routinely cultured with DMEM (HyClone, Logan, UT, USA) containing 20% fetal bovine serum (BI company, Petah Tikva, Israel), whereas NCM460 cells were cultured in DMEM containing 10% fetal bovine serum, and both media containing 100 units/mL of penicillin and 100 μg/mL of streptomycin. Cells were cultured in a humidified cell culture incubator at 37 °C containing 5% CO_2_.

### 2.3. Cell Viability Assay

Caco-2 and NCM460 cells were plated (4000 cells/well) in 96-well plates, polydextrose, fibersol-2, or fermentation broth of *F. nucleatum* at the designed concentration was added, respectively, and further cultured for 72 h. MTT (KeyGEN BioTECH, Nanjing, China) was used to assay the cell viability by Spark multifunctional microplate reader (Tecan AG, Männedorf, Switzerland).

### 2.4. Total RNA Extraction, Reverse Transcription, Quantitative PCR Assay

After the cells seeded at 5 × 10^5^ cells/well in 6-well tissue culture plates were cultured overnight, 2.5%, 5%, and 10% fermentation broths of *F. nucleatum* were added, respectively. The total RNA was extracted (Total RNA extraction kit, Magen, Guangzhou, China) 3 h later. A total of 1 µg total RNA was used for reverse-transcription using Fast Quant cDNA first-strand synthesis kit (Tiangen, Beijing, China) after assayed by Nano-drop (Thermo Scientific, Waltham, MA, USA).

Hieff UNICON^®^ qPCR SYBR Green Master Mix (Yeasen Biotechnology, Shanghai, China) was used for quantitative PCR and the program as follows: pre-denaturing: 95 °C, 3 min; a total of 40 cycles of 95 °C 10 s, 60 °C 20 s, and 72 °C 20 s, then melting curve analysis at the end. The relative gene expression was calculated by 2^−ΔΔCT^ method [53], and *GAPDH* was used as a reference.

Quantitative PCR gene primers were designed by Primer3 [54] and synthesized by Tsingke (Shanghai, China). The sequences of primers are listed in Table 1.

### 2.5. GC-Mass Spectrometry Analysis

Butyrate was analyzed GC-MS-MS (TSQ 8000, Thermo Fisher, USA) using the method reported [55] with some modifications. A total of 1000 µL of the supernatants of broth of *F. nucleatum* were used to extract butyrate and derivatized using 2,3,4,5,6-Pentafluorobenzyl bromide (PFBB) and N, N-diisopropylethylamine 100% (EDIPA) method [56]. The standard butyrate with known concentration was purchased from Sigma-Aldrich to establish a standard curve. The GC-MS analysis program was as follows: column flow rate: 1 mL/min, inlet and detector temperatures: 280 °C, 1 μL sample was injected by splitless injection; the oven temperature was initially maintained at 50 °C for 1 min and then increased to 280 °C at a rate of 10 °C/min. Helium was used as the carrier gas.

### 2.6. Phylogenic Analysis of Butyrate Biosynthesis Pathways

Genomes of *F. nucleatum* ATCC 25586 (GCA_000007325.1) and several other pathogenic (*Megasphaera elsdenii* DSM 20460 (GCA_000283495.2), *Flavonifractor plautii* YL31 (GCA_001688625.2), *Costridioides difficile* 630 (GCA_000009205.2), *Clostridium perfringens* ATCC 13124 (GCA_000013285.1), *Butyricimonas virosa* FDAARGOS 1229 (GCA_016889065.1) and *Porphyromonas gingivalis* ATCC 33277 (GCA_000010505.1), and non-pathogenic bacteria (*Faecalibacterium prausnitzii* L2-6 (GCA_000210735.1), *Coprococcus catus* GD/7 (GCA_000210555.1), *Roseburia intestinalis* M50/1 (GCA_000209995.1), *Eubacterium rectale* ATCC 33656 (GCA_000020605.1), and *Coprococcus eutactus* ATCC 27759 (GCA_020735705.1)) were analyzed by KEGG [57,58] to identify butyrate biosynthesis pathways. The key homologous genes (Table 2) existing in all species mentioned above harboring specific pathways were selected to construct the phylogenetic trees using a neighbor-joining algorithm in MEGA11 [59], with 1000 bootstraps testing.

### 2.7. Statistical Analysis

All results were presented as the mean ± SD calculated from three independent experiments. The data analysis employed various statistical tests depending on the distribution of the data. Parametric test such as one-way ANOVA was used when the data exhibited a normal distribution, while non-parametric tests, including the Kruskal–Wallis test and Bonferroni post-hoc test were utilized for data that did not follow a normal distribution. Statistical significance was determined at a *p*-value of less than 0.05. The statistical analysis was conducted using SPSS (version 22.0).

## 3. Results

### 3.1. Polydextrose and Fibersol-2 Did Not Affect the Growth of F. nucleatum Significantly

**Figure 1:**
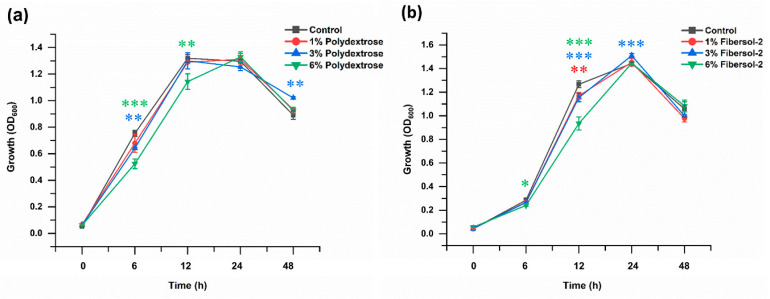
Effects of polydextrose and fibersol-2 on the growth of *F.nucleatum* ((**a**): Polydextrose, (**b**): Fibersol-2). Non-parametric Kruskal–Wallis test was used to calculate statistical significance. The red, blue, and green asterisks indicate statistical significance of 1%, 3%, and 6% experimental groups compared to the control, respectively. Note: *: *p* < 0.05; **: *p* < 0.01; ***: *p* < 0.001.

As illustrated in Figure 1a, although *F. nucleatum* grew 14.09% (*p* = 0.001) and 30.40% (*p* < 0.0001) slower than control after 3% and 6% polydextrose were supplemented and cultured 6 h; at 12 h time point, 13.34% (*p* = 0.009) growth retardation was identified only in 6% group; however, no further significant difference was observed at 24 h. Interestingly, the 3% group overgrew the control group by 14.56% (*p* = 0.002) at 48 h. When fibersol-2 was supplemented (Figure 1b) at the 6 h time point, 15.51% (*p* = 0.01) growth retardation was identified only in the 6% group; 7.68% (*p* = 0.004), 8.84% (*p* = 0.0002), and 26.18% (*p* < 0.0001) growth retardation was identified in 1%, 3%, and 6% groups at 12 h, respectively; however, at 24 h, only 3% group outgrew the control group by 5.54% (*p* = 0.0005), then no further significant difference was observed at 48 h.

### 3.2. Fermentation Broth of F. nucleatum Did Not Affect the Growth of Cells but Significantly Up-Regulated Proinflammatory Genes Expression in Caco-2 Cells

**Figure 2:**
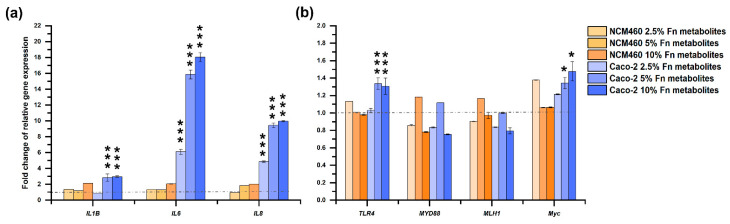
Effects of fermentation broth of *F. nucleatum* on selected genes expression in NCM460 and Caco-2 cells ((**a**): Polydextrose, (**b**): Fibersol-2). One-way ANOVA was used to calculate statistical significance. Note: *: *p* < 0.05; ***: *p* < 0.001.

Though no effects on the viability of both Caco-2 and NCM460 cells were observed when up to 10% final concentration of *F. nucleatum* fermentation broth was added and cultured for up to 96 h; however, as shown in Figure 2a, the expression of inflammatory cytokine *IL1B* in Caco-2 cells was significantly up-regulated to 184.87% (*p* < 0.0001) and 197.57% (*p* < 0.0001) in 5% and 10% supplement group; *IL6* and *IL8* were also significantly increased 511.91% (*p* < 0.0001), 1486.53% (*p* < 0.0001), and 1704.51% (*p* < 0.0001); 387.30% (*p* < 0.0001), 845.33% (*p* < 0.0001), and 897.05% (*p* < 0.0001) for 2.5%, 5%, and 10% groups, respectively. Likewise, a significant increase of 33.65% (*p* < 0.0001) and 30.70% (*p* < 0.0001); 34.37% (*p* = 0.018) and 47.67% (*p* = 0.021) was observed in the expression of *TLR4* and *Myc* for 5% and 10% groups, respectively, but no significant changes were detected for *MYD88* and *MLH1* in any group (Figure 2b). On the contrary, no significant expression changes in any gene mentioned above were detected when NCM460 cells were treated as same (Figure 2).

### 3.3. The Growth of Caco-2 Cells Was Significantly Suppressed by Fermentation Broth of F. nucleatum Cultured in Media Supplemented with Polydextrose or Fibersol-2

**Figure 3:**
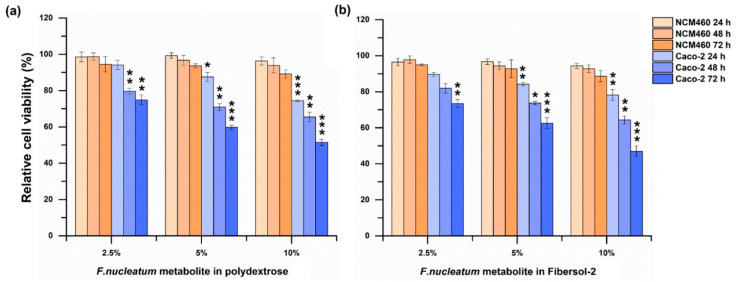
Effects of fermentation broth of *F. nucleatum* cultured in media supplemented with dietary fibers on the growth of NCM460 and Caco-2 cells ((**a**): Polydextrose, (**b**): Fibersol-2). One-way ANOVA was used to calculate statistical significance. Note: *: *p* < 0.05; **: *p* < 0.01; *** *p* < 0.001.

No significant effects were observed on the viability of both Caco-2 and NCM460 cells when up to 5% of polydextrose or fibersol-2 was added into the cell’s culture media for up to 72 h. As shown in Figure 3a, when the fermentation broth of *F. nucleatum* culture media supplemented with polydextrose, was added to Caco-2, for the 2.5% group, the relative survival rates at 48 and 72 h were considerably decreased by 21.32% (*p* = 0.002) and 25.09% (*p* = 0.0082), respectively; the 5% group were significantly decreased by 13.48% (*p* = 0.0170), 30.11% (*p* = 0.001), and 41.21% (*p* = 0.0004), respectively at 24, 48, and 72 h, and were noticeably decreased by 25.56% (*p* = 0.0001), 34.53% (*p* = 0.0013), and 48.90% (*p* = 0.0003), respectively for the 10% group. As shown in Figure 3b, for fibersol-2, Caco-2 cells’ relative survival rates were decreased by 11.38% (*p* = 0.01), 18.04% (*p* = 0.026), and 26.56% (*p* = 0.001), respectively at 24, 48, and 72 h in 2.5% group; the considerable decline of 15.80% (*p* = 0.0017), 27.30% (*p* = 0.0027), and 38.42% (*p* = 0.0009), respectively was observed in 5% group; the cell survival rate of the 10% group was remarkably declined 21.85% (*p* = 0.0052), 35.66% (*p* = 0.0018), and 52.96% (*p* = 0.0002), respectively.

Interestingly, no significant changes in cell survival were observed when NCM460 cells were treated accurately in the same manner (Figure 3).

### 3.4. Both Fibers Significantly Promote F. nucleatum-Producing Butyrate

**Figure 4:**
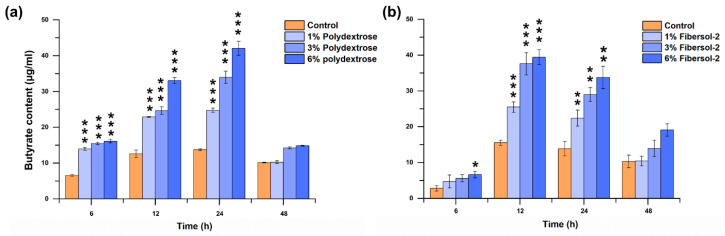
Effects of polydextrose and fibersol-2 on butyrate production of *F. nucleatum* ((**a**): Polydextrose, (**b**): Fibersol-2). Non-parametric ANOVA with Post-hoc test (Bonferroni method) for polydextrose and one-way ANOVA for fibersol-2 were used to calculate statistical significance. Note: *: *p* < 0.05; **: *p* < 0.01; *** *p* < 0.001.

As Figure 4a demonstrated, compared with the control, the production of butyrate was significantly increased by 112.68% (*p* < 0.0001), 135.62% (*p* < 0.0001), and 145.37% (*p* < 0.0001) at 6 h, 81.96% (*p* < 0.0001), 95.81% (*p =* 0.0009), and 162.53% (*p* < 0.0001) at 12 h, and 81.00% (*p* < 0.0001), 146.75% (*p* = 0.0002), and 205.67% (*p* < 0.0001) at 24 h, respectively, when the *F. nucleatum* was cultured in the media supplemented with 1%, 3%, and 6% of polydextrose correspondingly. In case of fibersol-2 (Figure 4b), although at 6 h, the significant increase of 135.43% (*p* = 0.026) for butyrate was only observed in 6% group, a significant increase of 63.96% (*p* = 0.00062), 141.77% (*p* < 0.0001), and 153.46% (*p* = 0.0002) at 12 h, and 61.23% (*p* = 0.0074), 108.89% (*p* = 0.019), and 143.16% (*p* = 0.0015) at 24 h was observed in 1%, 3%, and 6% groups, respectively. However, when *F. nucleatum* grew into the stationary phase after being cultured for 48 h, the butyrate production declined considerably, and no significant differences were detected in any groups mentioned above for both fibers.

### 3.5. Phylogenetic Analysis of Butyrate Biosynthesis Pathways among Selected Species

As shown in Table 3, only pyruvate and glutarate pathways were identified in all the five beneficial bacteria analyzed; however, only pyruvate and 4-aminobutyrate pathways were identified in the pathogenic ones, among these *B. virosa* and *P. gingivalis* harbored lysine pathways as well. The two opportunistic pathogens harbored three pathways, but the lysine pathway was absent in *M. elsdenii* and the 4-aminobutyrate pathway in *F. plautii*. Notably, only *F. nucleatum* harbored all four butyrate biosynthesis pathways among all the bacteria analyzed.

**Figure 5:**
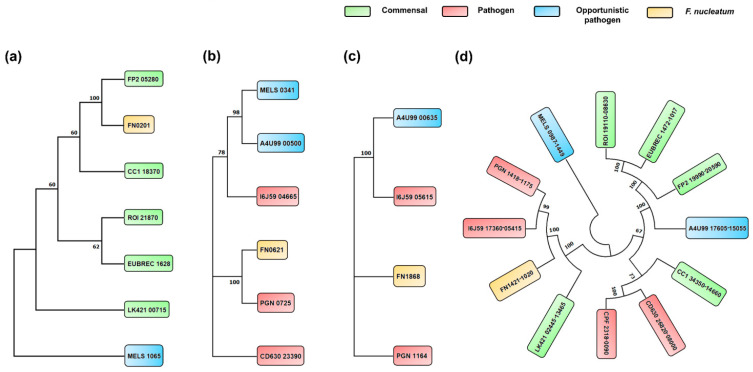
Phylogeny trees of four butyrate biosynthesis pathways for selected species ((**a**): Glutarate, (**b**): 4-aminobutyrate, (**c**): lysine, and (**d**): Pyruvate). Note: Green: commensal; blue: opportunistic pathogen; red: pathogen; yellow: *F. nucleatum*.

In the phylogenetic analysis, the Glutaconyl-CoA decarboxylase (Gcd) of *F. nucleatum*, which is crucial in the glutarate pathway, was clustered with that of commensal bacteria, and showed significant homology, particularly with *F. prausnitzii*, rather than clustering with the opportunistic pathogenic bacteria (*M. elsdenii*) (Figure 5a). The 4-aminobutyrate pathway exhibited two prominent clades, both harboring pathogenic bacteria and the 4-hydroxybutyrate coenzyme A transferase (4Hbt) of *F. nucleatum* was clustered with and displayed the highest similarity to the periodontal pathogen, *P. gingivalis* within the second clade (Figure 5b). In the phylogenetic analysis of the Lysine pathway, a similar pattern to the 4-aminobutyrate pathway was observed, and the 3-keto-5-aminohexanoate cleavage enzyme (Kce) of *F. nucleatum* clustered with that of pathogenic bacteria and showed the highest similarity to the periodontal pathogen, *P. gingivalis*, once again (Figure 5c).

Interestingly, for the pyruvate pathway (Figure 5d), the phylogenetic tree constructed based on the pyruvate-flavodoxin oxidoreductase (Por) and enoyl-Coenzyme A (CoA) hydratase (Crt) genes demonstrated that commensal, opportunistic, and pathogenic bacteria tended to exhibit higher similarity within their respective groups, but a clear boundary could not be identified. Notably, *F. nucleatum* was positioned in a sub-clade between pathogenic (*P. gingivalis*, *B. virosa*) and nonpathogenic bacteria (*C. eutactus*).

**Figure 6:**
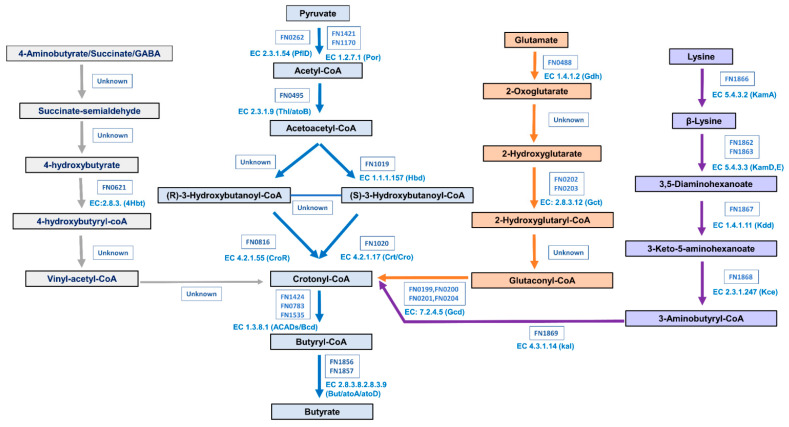
Schematic illustration of four butyrate biosynthesis pathways identified in *F. nucleatum*.

From the genome of *F. nucleatum*, beginning with the conversion of pyruvate (FN0262 (pfID), FN1421, and FN1170 (Por)) to Acetyl-CoA (FN0495 (Thl/atoB)), Acetoacetyl-CoA (Hbd (FN1019), (R) and (S)-3-Hydroxybutanoyl-CoA (CroR (FN0816), cro (FN1020)), and Crotonyl-CoA (Bcd (FN1424, FN0783, and FN1535) to yield Butyryl-CoA; from Butyryl-CoA through the butyryl-CoA, the acetate CoA transferase (But: atoD/atoA) route (FN1856 and FN1857) finally yielded butyrate, and almost all the genes involved were identified, which constituted the central pipeline of pyruvate pathway. Identification of the pyruvate pathway in *F. nucleatum* provided additional evidence and explanation regarding *F. nucleatum*’s ability to consume dietary fibers and enhance the butyrate production (Figure 6).

## 4. Discussion

For colon cancer, accumulating evidence proved that the consumption of processed meat as a high-risk factor and a fiber-rich diet as a beneficial factor could differentially modulate the gut microbiome structure [67,68], resulting in significant changes in the gut microbial metabolism [69], but the detailed mechanism has not been well elucidated so far. *F. nucleatum*, defined as one of the carcinogenic bacterium [70] by many studies, was proven as one of the lower risk factors of CRC in the study of a diet rich in dietary fibers [71]. In our study, no inhibitory effects of polydextrose and fibersol-2 on *F. nucleatum*’s growth were observed, consistent with several reports [71], and fibers even could significantly promote bacterial growth under specific conditions.

In this study, metabolites derived from *F. nucleatum* exhibited no inhibitory effects on the cell growth of the normal colon and CRC cell lines, except for a notable and exclusive up-regulation of expression of *TLR4* and *Myc* and genes encoding pro-inflammatory cytokines, including *IL1B*, *IL6*, and *IL8* in Caco-2 cells, when *F. nucleatum* cultured in normal BHI media. This finding was consistent with previous observations wherein *F. nucleatum* subsp. *polymorphum* was found to elicit a pro-inflammatory milieu through the activation of the TLR4 and subsequent NF-κB-mediated pro-inflammatory pathways [72].

For the pathways identified in *F. nucleatum*, such as lysine [45], glutarate [46,49] and 4-aminobutyrate [31,50] until now; the production of toxic byproducts, including ammonia, was reported [50] when *F. nucleatum* utilized amino acids to synthesize butyrate through 4-aminobutyrate and lysine pathways [73,74,75]. Notably, the presence of dietary fibers significantly facilitated *F.nucleatum* for the production of butyrate (205.67% increase, 6% polydextrose, 24 h), indicating the involvement of a complete pyruvate pathway, the primary pathway employed by bacteria to utilize dietary fiber for butyrate production [76,77], was identified in the genome of *F. nucleatum*. The aforementioned findings from this study provided evidence to elucidate why fermentation broth derived from fiber-supplemented groups markedly inhibited the growth of Caco-2 cells, while normal fermentation broth upregulated the expression of proinflammatory cytokines and the *TLR4* pathway at the transcriptional level. These findings suggested that the classification of *F. nucleatum* as a carcinogenic or commensal bacterium was determined by the dietary composition of the host.

Phylogenetic analysis (Figure 5) revealed that the homologous genes of *F. nucleatum* from the 4-aminobutyrate, lysine, and pyruvate pathways exhibited the highest similarity to the periodontal pathogen *P. gingivalis*. However, in the case of the glutarate pathway, the Gcd of *F. nucleatum* displayed the highest identity with that of *F. prausnitzii*. In terms of the pyruvate pathway, the only pathway existing in all bacteria analyzed, there was no clear separation between opportunistic and pathogenic bacteria, and *F. nucleatum* occupied an intermediate position between pathogenic bacteria (*P. gingivalis* and *B. virosa*) and nonpathogenic bacteria (*C. eutactus*). To explain why *F. nucleatum* exhibited a closer evolutionary relationship with the periodontal pathogen, *P. gingivalis*, rather than gut pathogens, our hypothesis was that *F. nucleatum* might be undergoing a transition from a periodontal bacterium [78] to a gut microbe.

In summary, dietary fibers played a pivotal role in determining whether *F. nucleatum* would inhibit the growth of CRC cells or activate the inflammatory signaling cascade through butyrate produced by *F. nucleatum*. The above findings indicated that *F. nucleatum* was likely the missing link between dietary composition, particularly the content of dietary fibers, and the risk of CRC occurrence and development. The detailed mechanism needs to be clarified before applicable suggestions for reducing CRC risk can be proposed. For example, how does *F. nucleatum* sense dietary fiber and activate the pyruvate pathway? Are there any other metabolites involved in it? What and how do dietary fibers affect the structure of gut microbiota mediated by *F. nucleatum*, since *F. nucleatum* plays a crucial role in shaping the bacterial community by affecting the growth of surrounding species [79,80]. Moreover, the effects of *F.nucleatum* and its metabolites on humans, especially the tumor initiation and progression-related pathways such as KRAS/BRAF, etc., should be investigated as well.

## Figures and Tables

**Table 1:** The primer sequences for RT-PCR.

Gene	Direction	Sequence (5′→3′)
*IL1B*	Forward	5′-AAACAGATGAAGTGCTCCTTCCAGG-3′
	Reverse	5′-TGGAGAACACCACTTGTTGCTCCA-3′
*IL6*	Forward	5′-CCCAGGAGAAGATTCCAAAGATGTA-3′
	Reverse	5′-GTCGAGGATGTACCGAATTTGTTTG-3′
*IL8*	Forward	5′-GAGAGTGATTGAGAGTGGACCAC-3′
	Reverse	5′-CACAACCCTCTGCACCCAGTTT-3′
*IL10*	Forward	5′-CCTGACCACGCTTTCTAGCTGTT-3′
	Reverse	5′-GGCTCCCTGGTTTCTCTTCCTAAG-3′
*TLR4*	Forward	5′-CATATCAGAGCCTAAGCCACCTCTC-3′
	Reverse	5′-AGCCACCAGCTTCTGTAAACTTGAT-3′
*MYD88*	Forward	5′-GAGGCTGAGAAGCCTTTACAGG-3′
	Reverse	5′-GCAGATGAAGGCATCGAAACGC-3′
*MLH1*	Forward	5′-TTGTTTTTATTGGTTGGATATT-3′
	Reverse	5′-AAATACCAATCAAATTTCTCAA-3′
*Myc*	Forward	5′-GAGGATCTCTGGGAGGAATGCTACT-3′
	Reverse	5′-CTGCTTTGTATTATCTGCGTGGCTA-3′
*GAPDH*	Forward	5′-TCCTGCACCACCAACTGCTT-3′
	Reverse	5′-GGGGCCATCCACAGTCTTCT-3′

**Table 2:** List of homologous genes existing in all selected species harboring specific pathways.

Species	Pathway	Representative Gene (Accession Number)	Description	References
*F. nucleatum* ATCC 25586	Glutarate	FN02019 (AAL94407)	Glutaconyl-COA decarboxylase beta subunit (GcdB)	[49]
	4- Aminobutyrate	FN0621 (AAL94817)	4-hydroxybutyrate coenzyme A transferase (4Hbt/ACH1)	[31]
	Lysine	FN1868 (AAL93967)	3-keto-5-aminohexanoate cleavage enzyme (kce)	[45]
	Pyruvate	FN1421 (AAL95614) FN1020 (AAL95216)	Pyruvate-flavodoxin/ferredoxin oxidoreductase (Por/nifJ) Enoyl-Coenzyme A (CoA) hydratase (Crt)	This study
*F. prausnitzii* L2-6	Glutarate	FP2_05280 (CBK98162)	Glutaconyl-CoA decarboxylase beta subunit (GcdB)	This study
	Pyruvate	FP2_19990 (CBK99399) FP2_20590 (CBK99451)	Pyruvate:ferredoxin (flavodoxin) oxidoreductase, homodimeric (Por), Enoyl-CoA hydratase/carnithine racemase (Crt)	[29]
*C. catus* GD/7	Glutarate	CC1_18370 (CBK80577)	Glutaconyl-CoA/methylmalonyl-CoA decarboxylase subunit delta (GcdD)	This study
	Pyruvate	CC1_34350 (CBK81960) CC1_14660 (CBK80244)	Pyruvate-ferredoxin/flavodoxin oxidoreductase (Por), Enoyl-CoA hydratase (Crt)	[31]
*R. intestinalis* M50/1	Glutarate	ROI_21870 (CBL09200)	Glutaconyl-CoA/methylmalonyl-CoA decarboxylase subunit delta (GcdD)	This study
	Pyruvate	ROI_19110 (CBL08968) ROI_08630 (CBL08094)	Pyruvate:ferredoxin (flavodoxin) oxidoreductase (Por/nifJ) Enoyl-CoA hydratase (Crt)	[60]
*E. rectale* ATCC 33656	Glutarate	EUBREC_1628 (ACR75372)	Pyruvate carboxylase subunit B (GcdC)	This study
	Pyruvate	EUBREC_1472 (ACR75226) EUBREC_1017 (ACR74779)	Pyruvate-ferredoxin/flavodoxin oxidoreductase (Por, nifJ) enoyl-CoA hydratase (Crt)	[61]
*C. eutactus* ATCC 27759	Glutarate	LK421_00715 (EDP27590)	Sodium ion-translocating decarboxylase subunit beta (GcdB/OAD_beta)	This study
	Pyruvate	LK421_02445 (EDP24924) LK421_13465 (EDP26522)	Pyruvate ferredoxin oxidoreductase beta subunit (PorB) Enoyl-CoA hydratase (Crt)	[62]
*M. elsdenii* DSM 20460	Glutarate	MELS_1065 (CCC73287)	Putative acetyl-CoA carboxylase biotin carboxyl carrier protein subunit (GcdC)	This study
	4- Aminobutyrate	MELS_0341 (CCC72564)	Acetyl-CoA hydrolase/transferase/4-hydroxybutyrate CoA-transferase (ACH1)	[63]
	Pyruvate	MELS_0987 (CCC73209) MELS_1449 (CCC73670)	Pyruvate-flavodoxin oxidoreductase (Por/nifJ), 3-hydroxybutyryl-CoA dehydratase (Crt)	[31]
*F. plautii* YL31	4- Aminobutyrate	A4U99_00500 (ANU39622)	4-hydroxybutyrate CoA-transferase (Cat2, AbfT ACH1)	[50]
	Lysine	A4U99_00635 (ANU39646)	3-keto-5-aminohexanoate cleavage protein (Kce)	[29]
	Pyruvate	A4U99_17605 (ANU42771) A4U99_15055 (ANU42306)	pyruvate:ferredoxin (flavodoxin) oxidoreductase (Por, nifJ) enoyl-CoA hydratase (Crt)	[64]
*C. difficile* 630	4- Aminobutyrate	CD630_23390 (CAJ69226)	4-hydroxybutyrate CoA transferase (Cat2, AbfT, ACH1)	[31]
	Pyruvate	CD630_26820 (CAJ69568) CD630_08000 (CAJ67634)	Pyruvate-ferredoxin oxidoreductase (Pfo/Por), 3-hydroxybutyryl-CoA dehydratase (Crt1)	[31]
*C. perfringens* ATCC 13124	Pyruvate	CPF_2318 (ABG84858) CPF_0090 (ABG84755)	Pyruvate-flavodoxin oxidoreductase (Por, nifJ) 3-hydroxybutyryl-CoA dehydratase (Crotonase) (Crt)	[31]
*B. virosa* FDAARGOS 1229	4- Aminobutyrate	I6J59_04665 (QRO51906)	Acetyl-CoA hydrolase/transferase family protein (ACH1)	[65]
	Lysine	I6J59_05615 (QRO51094)	3-keto-5-aminohexanoate cleavage protein (Kce/BKACE)	[66]
	Pyruvate	I6J59_17360 (QRO49639) I6J59_05415 (QRO51056)	Pyruvate:ferredoxin (flavodoxin) oxidoreductase (Por, nifJ), Short-chain-enoyl-CoA hydratase (Crt)	[66]
*P. gingivalis* ATCC 33277	4-Aminobutyrate	PGN_0725 (BAG33244)	4-hydroxybutyrate CoA-transferase (ACH1)	[50]
	Lysine	PGN_1164 (BAG33683)	3-keto-5-aminohexanoate cleavage enzyme (kce)	[50]
	Pyruvate	PGN_1418 (BAG33937) PGN_1175 (BAG33694)	Pyruvate-flavodoxin oxidoreductase (Por/nifJ) Putative enoyl-CoA hydratase (Crt)	[31]

**Table 3:** Association of butyrate biosynthesis pathway and pathogenicity among butyrate-producing bacteria analyzed.

Species	Pathogenic or Nonpathogenic	Pathway			
		Pyruvate	4-Amino Butyrate	Glutarate	Lysine
*F. nucleatum* ATCC 25586	Opportunistic pathogen	+	+	+	+
*F. prausnitzii* L2-6	Commensal	+	-	+	-
*R. intestinalis* M50/1	Commensal	+	-	+	-
*E. rectale* ATCC 33656	Commensal	+	-	+	-
*C. catus* GD/7	Commensal	+	-	+	-
*C. eutactus* ATCC 27759	Commensal	+	-	+	-
*M. elsdenii* DSM 20460	Opportunistic pathogen	+	+	+	-
*F. plautii* YL31	Opportunistic pathogen	+	+	-	+
*C. difficile* 360	pathogen	+	+	-	-
*C. perfringens* ATCC 13124	pathogen	+	+	-	-
*B. virosa* FDAARGOS 1229	pathogen	+	+	-	+
*P. gingivalis* ATCC 33277	pathogen	+	+	-	+

Note: -: absent; +: present.

## Data Availability

The data that support the finding of this study are available from the corresponding author upon reasonable request.

## Abbreviations

AtoB/Thl	Acetyl-CoA acetyltransferase / thiolase
Bcd	Butyryl-CoA dehydrogenase
But	Butyryl-CoA: acetate CoA transferase
But:atoD/atoA	Butyryl-CoA:acetate CoA transferase
Cro/Crt	Enoyl-CoA hydratase/crotonase
CroR	3-hydroxybutyryl-CoA dehydratase
Gcd	Glutaconyl-CoA decarboxylase
Gct	Glutaconate-CoA transferase
Gdh	glutamate dehydrogenase
4-Hbt	Butyryl-CoA:4-hydroxybutyrate-CoA transferase
Hbd	3-hydroxybutyryl-CoA dehydrogenase
Kal	3-aminobutyryl-CoA ammonia-lyase
Kam D, E	β-lysine-5,6-aminomutase, α/β subunit
KamA	Lysine 2,3-aminomutase
Kce	3-keto-5-aminohexanoate cleavage enzymes
Kdd	3,5-diaminohexanoate dehydrogenase
PflD	Formate acetyltransferase
Por	Pyruvate ferredoxin/flavodoxin oxidoreductase
